# Translation and validation of the Neurological Assessment in Neuro-Oncology scale to Brazilian Portuguese

**DOI:** 10.3389/fneur.2024.1369625

**Published:** 2024-06-25

**Authors:** Maíra Cristina Velho, Daniel Andrade Gripp, Paulo Henrique Pires de Aguiar, Joab Alves Nicacio, Cleiton Formentin, Gabriel Frizon Greggianin, Ana Carolina Pinheiro Campos, Marcos Vinicius Calfat Maldaun

**Affiliations:** ^1^Department of Neurosurgery, Clinical Hospital of Porto Alegre, Porto Alegre, Brazil; ^2^Neuro-Oncology Post-Graduation, Sirio-Libanes Hospital, São Paulo, Brazil; ^3^Department of Neurosurgery, Sirio-Libanes Hospital, São Paulo, Brazil; ^4^Department of Neurosurgery, Cristo Redentor Hospital, Porto Alegre, Brazil; ^5^Laboratory of Neuroscience, Sirio-Libanes Hospital, São Paulo, Brazil

**Keywords:** glioblastoma, brain metastasis, low-grade glioma, neuro-oncology, neurological performance, NANO scale

## Abstract

**Introduction:**

The Neurological Assessment for Neuro-Oncology (NANO) scale was elaborated to assess neurologic function in integration with radiological criteria to evaluate neuro-oncological patients in clinical setting and enable the standardization of neurological assessment in clinical trials. The objective of this study is the translation to Brazilian Portuguese and transcultural adaptation of NANO scale in patients with the diagnosis of glioblastoma, brain metastasis and low-grade glioma.

**Methods:**

Patients with diagnosis of glioblastoma, brain metastasis, and low-grade glioma were prospectively evaluated between July 2019 and July 2021. The process of translating and cross-culturally adapting the NANO scale included: translation from English to Portuguese, synthesis and initial revision by an expert committee, back-translation from Portuguese to English, a second revision by the expert committee, and the application of the NANO scale. Regarding the reliability of the NANO scale, Cronbach’s alpha was employed to measure the internal consistency of all scale items and assess the impact of item deletion. Additionally, Spearman’s correlation test was used to evaluate the convergent validity between the NANO scale and Karnofsky Performance Scale (KPS).

**Results:**

One hundred and seventy-four patients were evaluated. A statistically significant inverse relation (*p* < 0.001) between KPS and NANO scale was founded. The Cronbach’s alpha values founded for NANO scale were 0.803 for glioblastoma, 0.643 for brain metastasis, and 0.482 for low grade glioma.

**Discussion:**

The NANO scale Brazilian Portuguese version proves to be reproducible and valid to evaluate neuro-oncological patients with glioblastoma and brain metastasis, presenting a strong correlation with KPS scale. Further studies are warranted to assess the validity and reliability of the scale in patients diagnosed with low-grade glioma.

## Introduction

Central nervous system (CNS) tumors are responsible for 1.5% of all cancers and for 2.4% of all cancer deaths annually (1). In Brazil, the latest estimative predicted around 11,000 new cases of primary CNS tumors for 2020 (2). In the last years, a constant increase in studies evaluating new treatments in neuro-oncology, such as immunotherapy and target therapy, showed the necessity of a more precise response assessment in clinical trials and clinical practice. However, the best way to establish the evaluation criteria is not well defined in literature. While overall survival is considered an objective parameter and a gold standard in most studies, this outcome is limited to evaluate response assessment in diseases with prolonged course, as observed in low grade gliomas or meningiomas. Therefore, it is essential the implementation of response criteria that include both clinical and radiological findings, to promote a more complete evaluation (3, 4).

Even with the well stablished radiological response evaluation proposed by RANO group (4–7), the neurological outcome incorporated is simplified as “better,” “stable” or “worse.” The usual measurement of functional and clinical outcome in the oncological clinical practice and trials is by the Karnofsky Performance Status (KPS) (8) and Eastern Cooperative Oncology Group Performance Status (ECOG) (9). Despite the easy applicability in clinical trials and practice, the main limitation of these instruments in the neuro-oncologic patient is the inefficacy to translate the neurological status even with its direct influence in functional performance. Additionally, functional deterioration does not always signify disease progression. Prolonged use of corticosteroids, chemotherapy, immunotherapy, radiotherapy, infections, pre-existing comorbidities, and psychiatric conditions are examples of potential confounders in the functional evaluation of neuro-oncologic patients.

In 2017, a multidisciplinary international committee composed by neuro-oncology experts developed the Neurological Assessment in Neuro-Oncology (NANO) scale and evaluates neurological function in neuro-oncologic patients. The primary goal of NANO is to define clinical parameters objectively, to measure clinical response and disease progression related to tumoral activity. This scale was developed to be a simple, easy and fast-to-use tool that can be employed by any healthcare professional. To maintain its objectivity, the evaluation is based in direct observation rather relying on clinical history or reported symptoms (10).

Instruments that quantify and stratifies variables are essential to reproduce new data and aggregate more substantial information to evaluate outcomes and, finally, guide therapeutical decisions. Therefore, the translation and validation support the use of an instrument across different languages and cultures, standardizing the evaluation worldwide. The objective of this study is to translate and validate of the NANO scale to Brazilian Portuguese.

## Methods

Patients diagnosed with brain tumors (presumably glioblastoma, low-grade glioma and brain metastasis) were prospectively evaluated between July 2019 and July 2021 at Sirio-Libanes Hospital, including both outpatient clinics and hospital admissions. Eligible patients were 18 years of or older with the previously mentioned CNS tumors, providing informed consent.

This study protocol was approved by the Institutional Review Board (ID 1294). Each patient underwent a neurological evaluation by a senior author’s staff neurosurgeon. The translation process of NANO scale included the following steps: translation from English to Portuguese, synthesis and initial revision by an expert committee composed by the senior authors (M.V.C.M, M.C.V., D.A.G., J.A.N.J., C.F., and G.F.G.), back-translation from Portuguese to English, a second revision by the expert committee, and the application of the NANO scale.

The NANO scale is an instrument that evaluates neurological function through nine domains: gait, strength, ataxia, sensibility, visual field, facial paralysis, language, level of consciousness and behavior. The scale domains were selected according to the most common neurological findings in patients with brain tumors. Each domain has three or four score levels, varying between 0 and 2 or 0 and 3. The zero score indicates normal function, and the highest level (2 or 3) refers to the worst deficit in that domain. Also, the options “not evaluated” and “not assessed” were added in each domain. “Not evaluated” is scored when any neurologic deficit is not attributed to tumoral activity, such as change in medications (corticosteroid therapy, sedatives, antiepileptic drugs), comorbid events (metabolic encephalopathy, post-ictal state, stroke), when the evaluation of one domain is affected by another domain (ataxia is not scored because of leg weakness, for example). “Not assessed” is scored when the examiner omits the evaluation of a domain (10). In Table 1, the NANO scale is described.

**Table 1:** Neurological Assessment of Neuro-oncology (NANO) scale.

Domain	Score	Function
Gait	0	Normal
	1	Abnormal but walks without assistance
	2	Abnormal and requires assistance (companion, cane, walker, etc.)
	3	Unable to walk
		Not assessed
		Not evaluable
Strength	0	Normal
	1	Movement present but decreased against resistance
	2	Movement present but none against resistance
	3	No movement
		Not assessed
		Not evaluable
Ataxia (upper Extremity)	0	Able to finger to nose touch without difficulty
	1	Able to finger to nose touch but difficult
	2	Unable to finger to nose touch
		Not assessed
		Not evaluable
Sensation	0	Normal
	1	Decreased but aware of sensory modality
	2	Unaware of sensory modality
		Not assessed
		Not evaluable
Visual Fields	0	Normal
	1	Inconsistent or equivocal partial hemianopsia (≥ quadrantopsia)
	2	Consistent or unequivocal partial hemianopsia (≥ quadrantopsia)
	3	Complete hemianopsia
		Not assessed
		Not evaluable
Facial Strength	0	Normal
	1	Mild/moderate weakness
	2	Severe facial weakness
		Not assessed
		Not evaluable
Language	0	Normal
	1	Abnormal but easily conveys meaning to examiner
	2	Abnormal and difficulty conveying meaning to examiner
	3	Abnormal. If verbal, unable to convey meaning to the examiner. OR non-verbal (mute/global aphasia)
		Not assessed
		Not evaluable
Level of Consciousness	0	Normal
	1	Drowsy (easily arousable)
	2	Somnolent (difficult to arouse)
	3	Unarousable/coma
		Not assessed
		Not evaluable
Behavior	0	Normal
	1	Mild/moderate alteration
	2	Severe alteration
		Not assessed
		Not evaluable

Adapted with permission from ‘The Neurologic Assessment in Neuro-Oncology (NANO) scale: a tool to assess neurologic function for integration into the Response Assessment in Neuro-Oncology (RANO) criteria’ by Nayak et al. (10), licensed under Oxford University Press-Journals.

The descriptive analysis of the collected data included: age, gender, interview time (diagnosis or follow-up reviews), lesion location, treatment timing at the evaluation, KPS score, presence of seizures and corticosteroid use. The statistical analysis was conducted with SPSS® for Windows (SPSS Inc., Chicago, IL, USA). Categorical variables were presented as proportions and analyzed with Pearson’s Chi-square Test and Fisher’s Exact Test. The continuous variables were submitted to the Kolmogorov–Smirnov Test to verify the normal distribution. The comparison was conducted with Student T test (normally distributed data), Mann–Whitney U test and Kruskal-Wallis test (non-normally distributed data). The post-hoc Dunn test was performed when the null hypothesis was rejected in Kruskal-Wallis test. Concerning NANO scale confiability, Cronbach’s alpha was utilized to measure the internal consistency of all scale items and if item deleted. The correlation Spearman’s Test was used to evaluate the convergent validity between NANO scale and KPS.

## Results

**Figure 1:**
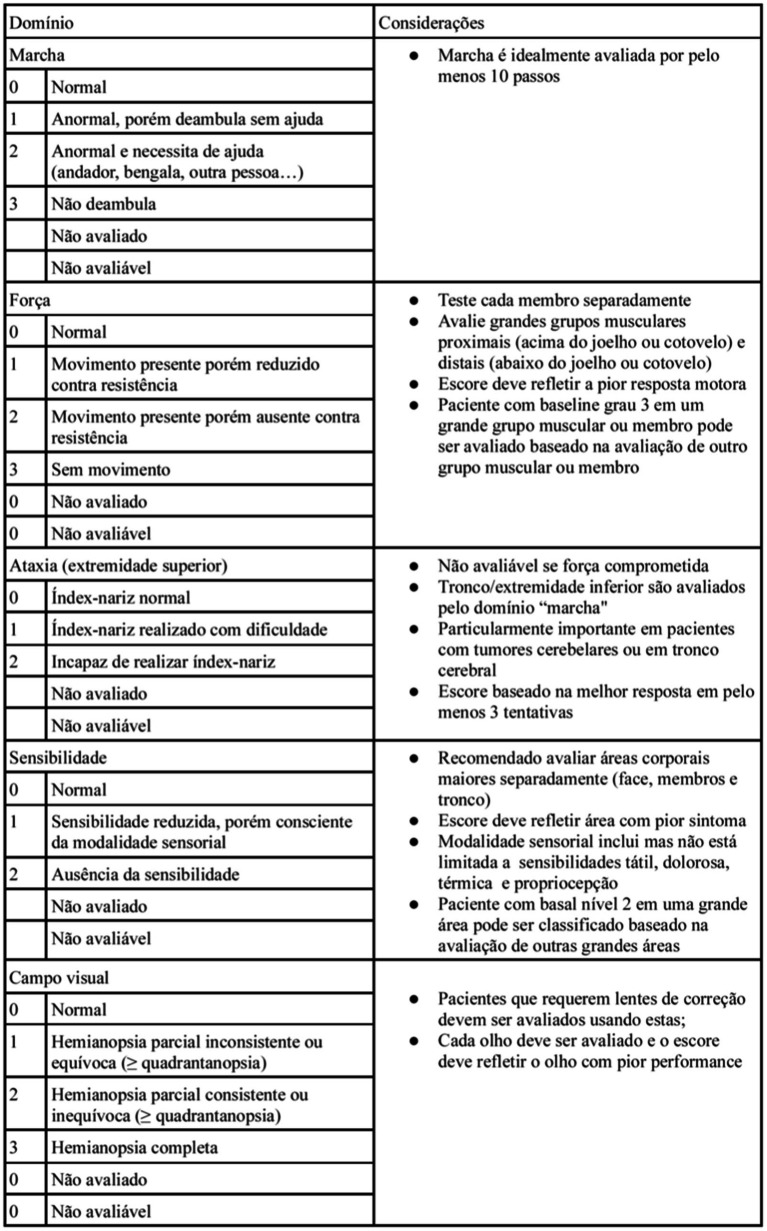

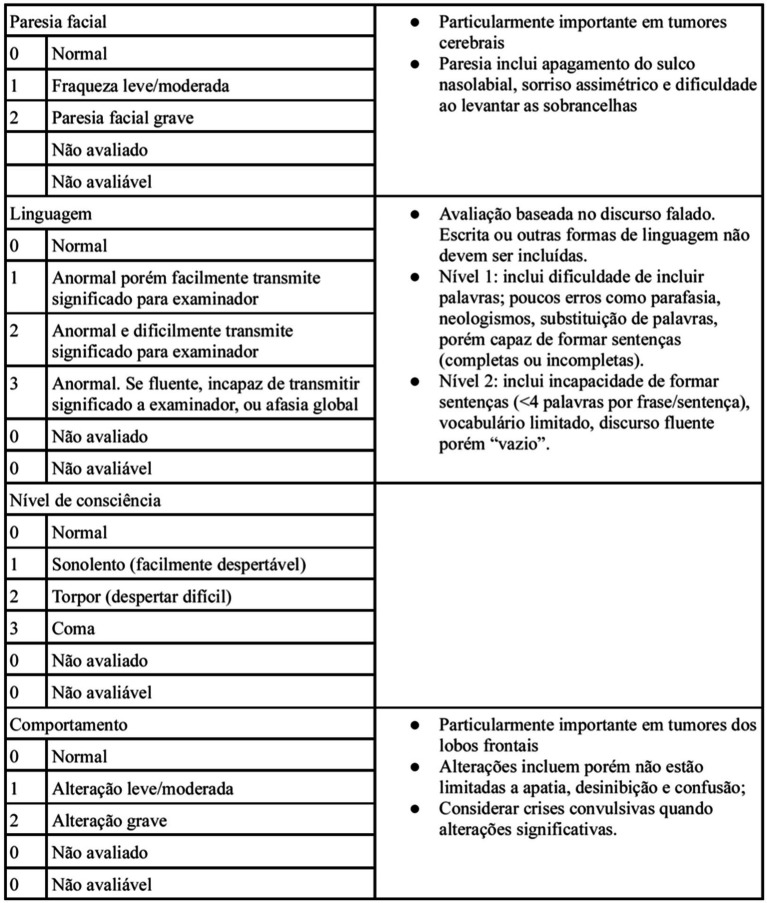
Final version of Brazilian translation of NANO scale.

In regarding to the NANO scale translation to Brazilian Portuguese process, no inconsistency was identified in all stages, including translation, initial revision, back-translation and its final version (the Brazilian Portuguese version is described in Figure 1).

After the translation process, 174 patients were evaluated. The epidemiological and clinical characteristics are presented in Table 2. There are no difficulties in the understanding of scale items among examiners. The option “not assessed” was not scored in any patient evaluated.

**Table 2:** Demographic and clinical characteristics.

	Disease (n)			*p*	Total
	Glioblastoma (76)	Metastasis (51)	Low grade glioma (47)		174
Age–median (P25-P75)	57 (42.7–64.2)	58 (43–66.5)	40 (34.5–46)	<0.001*†	
Gender				0.002**	
Female	33 (43.4%)	35 (68.6%)	16 (34%)		84 (48.3%)
Male	43 (56.6%)	16 (31.4%)	31 (66%)		90 (51.7%)
Moment of NANO application				0.644**	
At diagnosis	18 (23.7%)	10 (19.6%)	13 (27.7%)		41 (23.6%)
At follow-up	58 (76.3%)	41 (80.4%)	34 (72.3%)		133 (76.4%)
Localization					
Infratentorial	5 (7.6%)	10 (19.6%)	1 (2.1%)	0.015***	16 (9.8%)
Supratentorial	61 (92.4%)	41 (80.4%)	46 (97.9%)		148 (90.2%)
Moment of treatment				0.01**	
Chemotherapy	23 (32.9%)	10 (21.7%)	2 (4.3%)		35 (21.5%)
Radiotherapy	1 (1.4%)	2 (4.3%)	2 (4.3%)		5 (3.1%)
Chemotherapy and radiotherapy	5 (7.1%)	3 (6.5%)	3 (6.4%)		11 (6.7%)
No treatment	20 (28.6%)	5 (10.9%)	16 (34%)		41 (25.2%)
Other treatment	-	2 (2.3%)	1 (2.1%)		3 (1.8%)
Before surgery	13 (18.6%)	10 (21.7%)	10 (21.3%)		33 (20.2%)
After surgery	8 (11.4%)	14 (30.4%)	13 (27.7%)		35 (21.5%)
Seizures	35 (50%)	12 (26.7%)	30 (63.8%)	0.001**	77 (47.5%)
Corticosteroid use	34 (49.3%)	27 (58.7%)	6 (13.6%)	<0.001**	67 (42.1%)

* Kruskal-Wallis test.

** Pearson’s Chi-square test.

*** Fisher’s exact test extension.

† Post-hoc analysis (Dunn’s test). Differences between groups: glioblastoma and low-grade glioma (*p* < 0.001); low-grade glioma and brain metastasis (*p* < 0.001); glioblastoma and brain metastasis (*p* = 0.826).

### Convergent validity

**Figure 2:**
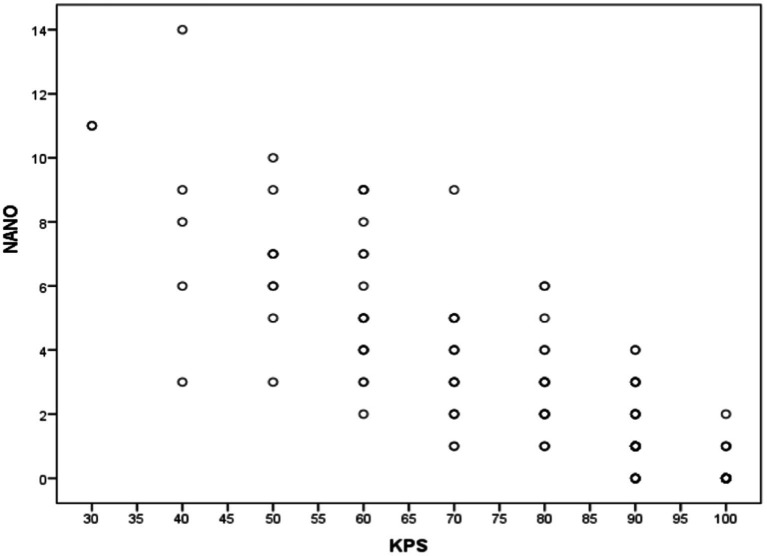
Spearman correlation coefficients between NANO and KPS scales.

**Figure 3:**
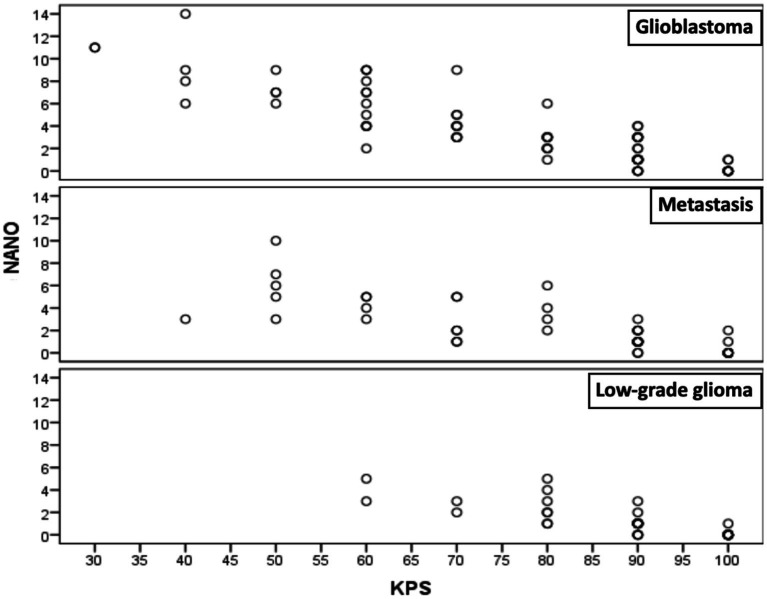
Spearman correlation coefficients between NANO and KPS scales in glioblastoma, brain metastasis and low-grade glioma groups.

Figure 2 specifies the total Spearman correlation coefficients between NANO and KPS scale. The value encountered (−0.875) was statistically significant (*p* < 0.001). Figure 3 shows the amplitude of the Spearman correlation coefficients founded between NANO and KPS scale in each disease. A significant inverse correlation (*p* < 0.001) was observed in all diseases. The coefficients were: *r* = −0.886 in glioblastoma, *r* = −0.827 in brain metastasis and *r* = −0.872 in low grade glioma.

### Confiability

The Cronbach’s alpha values for NANO scale founded for each disease is specified as follows: 0.803 for glioblastoma, 0.643 for brain metastasis and 0.482 for low grade glioma. The total Cronbach’s alpha is 0.777. In Table 3, detailed information about Cronbach’s alpha when items are deleted is provided. Differences between patient groups were identified. When the items “gait,” “strength,” “ataxia,” “facial paralysis,” and “behavior” were excluded, the Cronbach’s alpha dropped below the total value, indicating that these items are essential for the scale’s reliability. In regarding to the exclusion of “consciousness” item, higher scores were observed in all groups, which infers the reduction of scale reliability with the presence of this domain. The “visual field” and “language” items showed lower scores when deleted in the glioblastoma and brain metastasis groups. Finally, the “sensibility” domain was important only in the glioblastoma group.

**Table 3:** Total Cronbach’s alpha, by disease and if item excluded founded in NANO scale.

		Glioblastoma	Brain metastasis	Low-grade glioma
Total		0.803	0.643	0.482
If item excluded				
Item excluded	Gait	0.771	0.592	0.301
	Strength	0.784	0.594	0.342
	Ataxia	0.750	0.521	0.377
	Sensation	0.763	0.646	0.511
	Visual field	0.778	0.635	0.529
	Facial strength	0.798	0.616	0.398
	Language	0.794	0.618	0.533
	Level of consciousness	0.808	0.651	0.490
	Behavior	0.797	0.636	0.477

## Discussion

This was the first study of translation and cross-cultural validation of NANO scale into Brazilian Portuguese. The primary objective was to translate and validate the NANO scale into Brazilian Portuguese with glioblastoma, brain metastasis and low-grade glioma patients. We chose these diseases due to the potential variability of identified symptoms and to assess whether there are specific particularities in each disease that could impact the NANO scale approach.

The radiological response criteria have already been validated, and the RANO criteria and its offspring (RANO-LGG, RANO-HGG, RANO-BM) (4–7, 11) are widely used in clinical trials. However, the clinical criteria also included in those instruments are too simplified and are commonly associated with the KPS and ECOG/WHO scores, which reflect only the general functional condition, without providing further details on the neurological status. Besides, radiological parameters not always mirror the neurological status. The greatest examples are the pseudoprogression and pseudo response phenomena (4, 6, 12). It is essential to perform a combined evaluation of radiological and more specific clinical criteria to determine treatment response. The NANO scale effectively addresses the limitations of other scores by characterizing the clinical status through nine domains that can be quantified and assessed longitudinally. The scale is suitable for use by all professionals involved in patient care.

Although it is a novel instrument, the NANO scale has been utilized in various studies to date. These studies were heterogeneous, including patients with different diagnoses such as glioblastoma, low-grade glioma, meningioma, and neurological patients in general. The NANO scale has been employed in both prospective studies (13–17) and retrospective ones (18–23). Despite the need for NANO validation with retrospective data, there appears to be no significant loss of clinical information.

In Rahman et al. and Nayak et al., the NANO scale was used to measure neurological deficits in clinical trials in conjunction with the RANO criteria, following the approach proposed by the instrument’s authors (15, 16). Additionally, the NANO scale serves as an early predictor of disease progression in gliomas and a predictor of survival in glioblastoma when compared to other commonly used scales for assessing functional status. Good initial NANO and KPS scores predict 4–5 times better functional recovery 2 months after resection. Compared to KPS, the NANO scale exhibits a stronger correlation with better outcomes and is superior in predicting functional improvement (13). In a Korean study, 76 glioblastoma patients were retrospectively reviewed. In this study, the NANO scale demonstrated greater prognostic value at diagnosis and disease progression compared to the KPS and ECOG/WHO scales (21). In a retrospective study, a worse NANO score was the only metric associated with lower survival when compared to the RANO and MacDonald criteria at 6 and 12 months after treatment (23). As per Heiland et al.’s study, they assessed the functional response following surgical treatment in 342 elderly patients with glioblastoma. Patients with higher NANO scores were more likely to be submitted to biopsy than gross total resection (GTR: 2.57 vs. biopsy: 2.74; *p* < 0.05). In addition, lower NANO scores were significantly associated to longer survival (*p* = 0.001) (18). In Table 4, all the published studies that utilized the NANO scale are described.

**Table 4:** Summary of all studies published so far using NANO scale.

Study	Population	Utilization of another criteria	Observations
Heiland et al. (18)	Glioblastoma in elderly population	KPS	Retrospective study. Treatment should be decided considering the functional/neurological status, not only age.
Lee et al. (21)	Glioblastoma	KPS, ECOG	Retrospective study. NANO provides better objective measure of neurological status than other scales, especially in disease progression.
Ung et al. (23)	Glioblastoma	KPS, ECOG, RANO-HGG	Retrospective study. Only NANO scale was associated with 1-year overall survival. NANO score progression was the only metric related to decreased survival compared to RANO and MacDonald criteria in 6 and 12 months after diagnosis.
Gunawan et al. (13)	Glioblastoma, low-grade glioma	KPS	Prospective cohort. Patients with lower scores at diagnosis have 4–5 times more probability of better outcome.
Steindl et al. (22)	Neurologic patients	No	Retrospective cohort comparing healthy individuals vs. Patients with a neurological condition. The relation between temporal muscle thickness and strength is investigated, as a tool to evaluate sarcopenia. NANO scale was used to measure neurologic deficit.
Kong et al. (20)	Low-grade glioma and glioblastoma	No	Retrospective case series evaluating neuronal plasticity in glioma patients after surgery. NANO scale used to measure neurologic deficit before and after surgical treatment.
Rahman et al. (16)	Glioblastoma	No	Prospective IB trial evaluating the effect of bortezomib e temozolamide. NANO scale measured neurologic deficit in combination with RANO criteria.
Kalasauskas et al. (14)	Meningioma	No	Prospective study comparing the psychological burden in patients with the diagnosis of meningioma, submitted to surgery vs. conservative treatment.
Nayak et al. (15)	Glioblastoma	No	Prospective randomized phase II study comparing the use of pembrolizumab and bevacizumab vs. pembrolizumab. NANO was used to measure neurological deficits, assessed in conjunction with RANO criteria.
Kalasauskas et al. (24)	Meningioma	No	Prospective study comparing distress and quality of life in patients diagnosed with meningioma undergoing surgery vs. Conservative treatment. NANO score was not related to symptoms of anxiety, depression, or distress.
Kasper et al. (19)	Glioblastoma	No	Retrospective study evaluating IDH non-mutated glioblastoma at diagnosis, post-operative period and at 3 months follow-up.
Therkelsen et al. (17)	Primary central nervous system lymphoma	KPS	Prospective case series evaluating patients submitted to chemotherapy with autologous hematopoietic stem cell transplant for Primary central nervous system lymphoma.

As presented in the NANO validation publication (10), the option ‘not assessed’ was included in all domains. This option applies to situations in which the interviewer deliberately does not assign any scores to a particular domain, and subsequently, that domain should be excluded from further evaluations. However, during data collection, we observed that such circumstances did not arise. This observation suggests that the option ‘not assessed’ may be omitted in all domains in future studies. Few patients received ‘not evaluated’ scores, and as a result, it cannot be determined if this has a considerable influence on the final score. Additionally, this option was excluded in the evaluation between the groups.

Since this is the first instrument specifically designed to evaluate neurological status in neuro-oncological patients, there is no established “gold-standard.” Therefore, we used the convergent correlation with KPS score in the validation process, given its widespread application in neuro-oncology. A significant inverse correlation (*p* < 0.001) was found between the scales in all groups (glioblastoma: *r* = −0.886; metastasis: *r* = −0.827; low grade glioma: *r* = −0.872). Consequently, the higher the KPS score, the lower NANO score will be. To date, there are no published studies correlating the NANO with other instruments (13, 18, 21, 23).

Among the diverse ways to measure the reliability, we used the internal consistency, applying Cronbach’s Alpha as the statistical test. The internal consistency varied between groups: in the glioblastoma and metastasis patients, the Cronbach’s Alpha were acceptable (0.803 and 0.643, respectively). However, the Cronbach’s Alpha found in the low-grade glioma group was 0.482, which is an insufficient coefficient result. This lower value may be explained by the longer disease duration and milder signals and symptoms severity and variability compared to glioblastoma and brain metastasis, which can impair the scale’s evaluation in this specific group. It is possible that with a larger sample size, we may achieve better results in terms of internal consistency in this specific population.

In relation to Cronbach’s Alpha if an item is deleted, differences were observed between groups. This analysis allows us to assess the influence of each domain on the instrument’s internal consistency. If Cronbach’s Alpha decreases when an item is deleted, it indicates that this domain is important for the instrument’s reliability (25). As specified in Table 2, excluding “gait,” “strength,” “facial paralysis” and “behavior” domains decrease the Cronbach’s Alpha coefficient, indicating their key role in the instrument’s reliability. This finding may be explained by the higher objectivity and consistency in clinical evaluation, as well as their frequency, which allows for better internal consistency evaluation. Cronbach’s Alpha increases when this item is excluded, suggesting a reduction in reliability with the presence of this domain. However, this data may suggest the possibility of excluding this item to improve the scale’s performance. Nevertheless, since this is a novel instrument with recent applications, further studies are needed to confirm the significance of this item and the variability observed between groups, as shown in Table 3.

The NANO scale was developed to be applied alongside radiological criteria, providing a comprehensive evaluation and being useful for the follow-up of neuro-oncological patients, regardless of tumor histology. However, understanding the particularities found in each subpopulation is essential for refining the instrument and maximizing its utility in neuro-oncology. For instance, excluding the NANO validation publication (10), the instrument has not yet been applied to patients with cerebral metastasis. This measurement could be important since the assessment of clinical status significantly influences therapeutic decisions. For example, asymptomatic patients may undergo systemic treatments with good central nervous system penetrance, while those with focal symptoms could be treated with more localized therapies like surgery or stereotactic radiosurgery. Furthermore, with better quantification and monitoring of neurological symptoms, patients with brain metastasis have a greater chance of being included in clinical trials, a condition that is typically an exclusion criterion in studies (26).

The NANO scale is an instrument applied by healthcare professionals, with domains characterized by technical terms widely used in clinical practice, without expressions or terms that can lead to misunderstanding or the necessity to transcultural adaptation. Due to these factors, there were no disagreements in none of translation steps. The instrument’s application did not require prior training, and the provided instructions were sufficient for its execution. These characteristics encourage its use in clinical practice, promoting better communication among healthcare professionals.

### Study limitations

The interobserver agreement could not be assessed due to an insufficient sample size required for this analysis. We plan to address this reliability analysis in future studies. The score ‘not evaluated’ was identified in a few cases; therefore, it was not possible to assess its influence on the validity and reliability of the NANO scale. The Cronbach’s alpha found in the low-grade glioma group was lower than recommended, and it was not possible to ensure the reliability of the NANO scale in this population. However, we intend to expand our sample size in future studies to provide better data for this specific population.

## Conclusion

The NANO scale is a relatively new instrument for neuro-oncology evaluation. Consequently, the validation process is crucial to assess its ability to reliably reflect the patient’s neurological status. This instrument is not limited to one-time evaluations; rather, it can detect neurological changes throughout the clinical course of the disease. The Brazilian Portuguese version of the NANO scale proves to be a reproducible and valid tool for evaluating neuro-oncological patients, demonstrating a strong correlation with the KPS scale and adequate overall internal consistency. However, further studies are required to assess its reliability in patients with low-grade glioma, despite its good correlation with the KPS score.

## Data availability statement

The raw data supporting the conclusions of this article will be made available by the authors, without undue reservation.

## Ethics statement

The studies involving humans were approved by Institutional Review Board of Sirio-Libanes Hospital. The studies were conducted in accordance with the local legislation and institutional requirements. The participants provided their written informed consent to participate in this study.

## Author contributions

MV: Writing – review & editing, Writing – original draft, Visualization, Validation, Project administration, Methodology, Investigation, Formal analysis, Data curation, Conceptualization. DA: Writing – review & editing, Investigation, Data curation. PP: Writing – review & editing, Supervision. JN: Writing – review & editing, Validation, Methodology, Investigation, Data curation. CF: Writing – review & editing, Validation, Supervision, Methodology, Investigation, Formal analysis. GG: Writing – review & editing, Investigation, Data curation. AC: Writing – review & editing, Methodology. MM: Writing – review & editing, Writing – original draft, Supervision, Project administration, Investigation, Data curation, Conceptualization.
